# Graduate Study on the Continent—Germany

**Published:** 1908-07-18

**Authors:** 


### HOSPITAL ADMINISTRATION.

### CONSTRUCTION AND ECONOMICS.

### GRADUATE STUDY ON THE CONTINENT.—GERMANY.

VIII.—SOME OTHER GERMAN UNIVERSITIES.

In the last article we have briefly outlined the main points regarding the smaller German University towns that offer attractions to the foreign graduate. It remains to consider still the larger centres, which in many ways offer as varied attractions as Berlin. As a matter of fact, the largest medical University in Germany is not Berlin, but Munich, while Bonn and Leipsic rival the capital in the number of their students and in the wealth and variety of their clinical material.

Bonn

The large University of Bon is one of the best known and most popular in Germany, although it is, from the medical graduate's point of view, less important than the other large centres. As a "graduate town" Bonn has much to commend it. Its social attractions are manifold and various, and its clinical institutions are up-to-date. Especially fine are the new laboratories for chemical and practical physiology, for topographical anatomy, pathology, and pharmacology. Among the Professors are Professors von Leydig, Ribbert (the pathologist) who is Dean of the Faculty), Pflüger (the physiologist), Valetta St. George (anatomy), Schultze (medicine), Nussbaum (histology), Westphal (psychiatry), and Kruse (bacteriology). The clinical institutions consist of the large and well-directed medical clinic, medical polyclinic, dermatological polyclinic, surgical clinic and polyclinics, gynæcological clinic and polyclinic, clinic for diseases of the eye, ear, nose, and throat, for nervous diseases, and a new second medical clinic. The material available for study is varied and excellent, and there is a fine University library, besides special medical collections available for the student engaged in research work. The courses that are given here during the vacations are good, and special courses can be arranged with the *docenten*. Not the least of the attractions of the town is its vicinity to the picturesque Rhineland districts, which permits of easy excursions to the neighbouring towns of Godesberg and Königswinter, or by the excellent steamboats of the Rhine Company to Cologne or down the river. At Cologne very good vacation courses are given at the recently established Academy of Medicine, where the clinical material is far in excess of that at Bonn, and where the graduate has the opportunity of studying fractures and their treatment under one of the foremost authorities on the subject, Professor Dr. Bardenheuer.

Breslau.

Probably no German University enjoyed the reputation which Breslau possessed as a centre for medical study during the *régime* of the late Professor von Mikulicz Radecki, and although "the master" is no longer there, the medical school is still (and justly) regarded as one of the finest in Germany. Clinically Breslau serves a very large and densely populated district, and patients crowd to it from all parts of the neighbouring Galician and Polish provinces. It possesses several excellent hospitals, and the University clinics, for all the departments in surgery and medicine (excluding orthopædics), are in many ways equally excellent. The difficulty the foreign graduate will encounter in his work here will lie mainly in the out-patient department, and will be due to the fact that so many of the patients speak only Polish. For that reason Breslau is not to be recommended to the "Auslander," except for a short visit to become acquainted with the methods. In surgery this is particularly desirable, as nowhere else (with the possible exception of Cracow) is the "Mikulicz school" so well represented. Living is cheap and good, though most of the houses are old, and devoid of the modern comforts which can be obtained elsewhere. During the vacation courses are given in a variety of subjects, both in German and in Polish.

Giessen.

This centre offers few opportunities for foreign graduates, while the medical faculty is comparatively a small one, and the facilities for clinical study are not to be compared with those of other towns. Like Rostock, however, it has the advantages which belong to the small centre, but no special courses are given here, while at Rostock a most excellent series of post-graduate courses is held every winter vacation. Living is very cheap, and is said to be as good as it is inexpensive, but we have no personal experience of Giessen, and cannot vouch for the truth of the statement. There is a large veterinary college attached to the Grand Ducal University, and the anatomical and pathological laboratories are new and good. A small but well-stocked University library exists.

Jena.

Though not by any means one of the oldest German Universities, the Grand Ducal University of Jena can pride itself on a memorable history; Schiller and Goethe have both been among its professors, and there was a time when Jena figured largely in the politics of Europe. Nor has Jena omitted to make its notch in the tally of medical history, and the faculty has numbered great and well-known names. To-day Jena is a quiet University town, in which the student element is conspicuous, though there is no longer the nightly street brawl or the concerted attack upon the policemen that made the Jena student notorious in times past. Foreign graduates are admitted as matriculated students on mere production of their diplomas. There are about 300 medical students, and the medical school is one of the best in Germany. The clinical material, however, is relatively small, and in many respects Jena may be compared with Göttingen. For the graduate who wishes to do private research work this University is to be commended, as it possesses a fine library and several good pathological collections. Among the best-known members of the medical faculty are Professors Müller (pathology), Riedel (surgery), Krause (medicine), von Bardeleben (anatomy), and Wagenmann (ophthalmology). Dr. Meyer gives a special course of lectures on the history of medicine. Very popular and well-attended graduate courses are given during July. It is a town thoroughly deserving the attention of the graduate who does not wish to do much practical work in surgery or medicine, for which the small amount of available clinical material hardly offers many opportunities.

Marburg.

Here, too, the medical faculty is a small one and the clinical material unimportant when contrasted with the richness of the larger centres. Marburg is essentially a small University town, and as such it has its attractions as well as its drawbacks. A guest-ticket which admits to all lectures and demonstrations is granted free of charge to all approved foreign graduates on written application to the dean of the faculty the student wishes to become attached to. Among the professors of the medical faculty are Excellenz von Behring (medicine), Profesor Küster (surgery), Ahlfeld (gynæcology), Sauerbruch (operative course in surgery), and Krauss (diseases of the eye).

Munster.

What has been said of Marburg and the smaller universities applies to this comparatively modern university. Guest-tickets are not usually given, but foreign graduates are inscribed on the lists of students on production of their diplomas and on payment of a fee of 15 marks. Graduates who merely wish to attend a few courses should apply to the professor for permission to attend. Such permission is usually readily granted, but does not entitle the student to attend other classes. Professors Salkowski (chemistry), König (hygiene), and Tobler (bacteriology), are among the professors on the medical faculty. The facilities for medical study are not to be compared with those at Marburg, but an attempt is being made to organise the clinical material. At present there are no medical students and no lectures are given in clinical subjects, though the instruction in preliminary subjects is good.

Erlangen.

This is another small university, but it possesses a well-organised medical school, among the professors of which are: Profesors Penzold (medicine), Gerlach (anatomy), Graser (surgery), Specht (psychiatry), and Fuchs (physiology). Lady graduates are admitted on an equality with male students. The clinics are excellent, and the courses are equal to those given in any large centre. Special graduate courses are given, for admission to which early application to the demonstrators is desirable. There are many oportunities for special work, and the graduate who wishes to work for a long time at any speciality will find few better places than Erlangen. Living expenses are slightly higher than at most other small university towns.

Tubingen.

Foreign graduates must produce their diplomas and show that they have passed an examination equivalent to that demanded of the German matriculates. Those unable to do so can only be inscribed as "extraordinary auditors." The clinics are excellent, and the vacation courses are usually very well attended. The medical faculty includes Professors von Bruns (surgery), Grützner (physiology), Baumgarten (pathology), Schleich (ophthalmology), Romberg (medicine), Wolf (hygiene), Vierordt (medicine—also a special course on the history of medicine), Heidenhain (osteology), and Gaupp (psychiatry). The clinical material is unusually rich and varied for a small university town, and the surgical and pathological student especially will find a time spent at Tubingen by no means wasted if he uses the many opportunities that are available.

Würzburg.

The medical school here is small but well organised. Unless the graduate wishes to work at a special subject in quiet seclusion, it is scarcely advisable for him to spend much of his time here, as the clinical material available is not very large, even for a small centre. Of the various clinics, the medical (Prof. Rindfleisch), the surgical (Prof. Rosenberger), and the special orthopædic (Prof. Riedinger) are well worth the student's attention.

